# Non-Invasive Autonomic Neuromodulation for Overactive Bladder: A Comparative Pilot Trial of NESA and Tibial Nerve Stimulation

**DOI:** 10.3390/jcm14248881

**Published:** 2025-12-16

**Authors:** Paloma M. Blasco-Bonora, Raquel Medina-Ramírez, Blanca Gisela Pardo-Sievers, Elena Muñoz-Gómez, Marta Inglés, Laura Fuentes-Aparicio

**Affiliations:** 1 Clínica de Fisioterapia L’estiuet, Carrera Malilla, 78, 46026 Valencia, Spain; centrofisioterapiaestiuet@gmail.com; 2 Soc-Dig Research Group, University of Las Palmas de Gran Canaria, 35001 Las Palmas, Spain; 3 University Clinical Hospital of Valencia, Av. de Blasco Ibáñez, 17, El Pla del Real, 46010 Valencia, Spain; bpardosievers@gmail.com; 4 Research Unit in Clinical biomechanics (UBIC), Department of Physiotherapy, Faculty of Physiotherapy, University of Valencia, 46010 Valencia, Spain; elena.munoz-gomez@uv.es (E.M.-G.); marta.ingles@uv.es (M.I.); 5 Physiotherapy in Motion, Multispeciality Research Group (PTinMOTION), Department of Physiotherapy, Faculty of Physiotherapy, University of Valencia, 46010 Valencia, Spain; laura.fuentes@uv.es

**Keywords:** overactive bladder, posterior tibial nerve stimulation, neuromodulation, transcutaneous electric nerve stimulation

## Abstract

**Objectives**: We aimed to evaluate the effect of non-invasive NESA neuromodulation compared to posterior tibial nerve stimulation (PTNS) in patients with an overactive bladder (OAB), also given the same exercises and patient education, on quality of life, symptoms, discomfort and sleep quality. **Method**: Twenty-four women, aged 38–85 years with OAB, were included in this preliminary randomized controlled trial. Each patient attended ten sessions, twice a week. Patient pelvic floor function and urinary incontinence symptoms were collected throughout ICIQ-SF and B-SAQ questionnaires. Patient QoL and sleep quality were reported using SF-36 and PSQI, respectively. All outcomes were measured using three assessments: previous treatment (T1), immediately after treatment (T2) and two-month follow-up (T3). **Results**: Both groups showed significant improvements in pelvic floor function and urinary incontinence symptoms, as well as in sleep quality (*p* < 0.05). Although no significant differences between the groups were observed for any of the variables (*p* > 0.05), only the NESA group showed compelling improvements in quality of life (*p* < 0.05). **Conclusions**: The two treatments improved OAB symptoms, discomfort, and sleep quality in the short term yet only the non-invasive NESA group improved quality of life in women with OAB. These findings warrant further investigation in larger trials.

## 1. Introduction

Overactive bladder (OAB) represents urinary dysfunction with the greatest impact on health, quality of life (QoL), anxiety, depression, and sleep among affected individuals [1,2]. Population studies indicate that the prevalence of urinary incontinence and OAB increases with age, ranging from 26.8% to 39.5% in men and women across Europe. Symptom presentation differs between sexes, being higher in women than in men, and increases with age [2]. In Spain, the prevalence of OAB approaches 10% in women aged 25–64, is around 5% in men aged 50–65 and exceeds 50% in individuals over 65 of both sexes. Among children aged 6–11 years, approximately 6% experience nocturnal enuresis, collectively resulting in a substantial economic burden [1].

The etiology and pathophysiology of OAB is multifactorial and may vary between individuals. The micturition reflex is triggered when the densely innervated detrusor muscle stretches, allowing synchronous activation and increased intravesical pressure. Bladder control is mediated by complex interactions between the central and peripheral nervous systems, including modulation of the autonomous nervous system (ANS) [3]. Partial pathological alterations of the detrusor, whether neurogenic, myogenic, or idiopathic or due to any imbalance in the excitation or inhibition of smooth muscle modulators, can lead to detrusor overactivity. This affects the bladder’s autonomous sensory pathway and contributes to the need to avoid at low volumes, resulting in urgent urinary incontinence [4].

The management of overactive bladder (OAB) follows a stepwise approach aimed at reducing urinary urgency, frequency, and incontinence, while improving patient quality of life. Treatment strategies can be broadly categorized into pharmacological treatment, conservative therapy and minimally invasive or invasive procedures. Pharmacological treatments, including antimuscarinics and β3-adrenoceptor agonists, are indicated since both have demonstrated efficacy in reducing urgency, frequency, and incontinence episodes, although antimuscarinics are often associated with higher rates of adverse events and treatment discontinuation. Conservative therapy, which includes lifestyle modifications and pelvic floor muscle training (PFMT), is preferred [5]. These strategies have been shown to improve muscle function and reduce urinary symptoms. Finally, minimally invasive or invasive procedures, such as sacral nerve stimulation (SNS), percutaneous PTNS, and onabotulinumtoxin A injections, are considered for patients with persistent symptoms. Despite conservative and pharmacological therapy and requiring patient commitment, they generally have acceptable safety profiles [6].

One of the first-line conservative interventions for urinary incontinence is therapeutic exercise. Specifically, in the case of an overactive bladder (OAB), pelvic floor muscle training (PFMT) has shown Level B evidence, with studies reporting reductions in urinary urgency and frequency. Combining PFMT with diaphragmatic exercises appears to improve muscle function and urinary symptoms more effectively than PFMT alone [5,6,7,8]. Further high-quality randomized controlled trials are needed to confirm these findings.

Besides physical exercise, there are currently several treatment options available for OAB, including electrical stimulation [9]. Posterior Tibial Nerve Stimulation (PTNS) is an effective, minimally invasive, and easily applied technique that is well tolerated by patients and recommended when first-line therapies fail [8]. PTNS (either percutaneous, PTNS, or transcutaneous, TTNS) has been confirmed to be an effective option for reducing daytime urination, episodes of urgency and nocturnal frequency in patients with idiopathic OAB, although with moderate quality evidence [10,11].

The combination of pelvic floor exercises with electrotherapy has yielded positive results for female sexual dysfunction (FSD), including arousal and orgasmic domain scores, and OAB [12]. A recent meta-analysis on pelvic floor dysfunction concluded that electrical stimulation combined with pelvic floor exercises increases pelvic muscle strength more than exercises alone (although without a clear improvement in QoL) [13,14].

It is important to note that although invasive devices can be effective, they present several disadvantages, including discomfort, embarrassment, the need to clean the probe, the risk of vaginal or urinary tract infections, difficulty handling the device, and user unwillingness to continue treatment [11,12,13]. In this context, minimally invasive and non-invasive approaches gain relevance, as they may be easier, more acceptable, and less embarrassing for patients. One such option is the administration of external electrical stimulation via surface electrodes placed on the skin, providing a more comfortable alternative with a lower risk of complications.

In the field of bladder dysfunction, new research fields are being explored regarding the use of non-invasive neuromodulation techniques [12] and devices as a tool to modulate the ANS and improve the symptoms. One of these emerging techniques is non-invasive neuromodulation NESA (applied surface neuromodulation) [15]. The NESA technology uses low-intensity, almost imperceptible, electrical microcurrents applied to low-impedance areas to stimulate and modulate the ANS [16]. Its aim is to regulate neural impulses to improve autonomic nervous system processing. This system is guided by Wilder’s law, which states that the body’s response depends on its previous functional state, and by the principle of hormesis, according to which small doses of a stimulus can produce beneficial or adaptive effects [15]. At a technical level, the electrical parameters used in NESA neuromodulation programs differ from those used in conventional TNS [11,17,18]. However, both currents are classified as low frequency. The NESA treatment is based on the application of a low-frequency biphasic current (between 1.14 and 14.28 Hz) where very low-intensity microcurrents (between 0.1 and 1 mA) are applied, with sequences designed to specifically modulate the ANS. These parameters are managed by the NESA neuromodulation device programs, except for voltage, which is set by the therapist to 3 or 6 volts [15]. Since emotional stress and depression can increase sympathetic activity and alter detrusor activity, the autonomic modulation of NESA could restore physiological balance and indirectly improve bladder function by reducing stress-related autonomic dysregulation. The NESA microcurrent has been proven to work in neurogenic bladder and pain in multiple sclerosis patients and in constipation in children [19,20]. However, there are no studies evaluating the effectiveness of NESA neuromodulation combined with therapeutic exercise programs in people with OAB.

The aim of this study was to investigate the effect of non-invasive NESA neuromodulation compared to posterior tibial nerve stimulation (PTNS), combined in turn with same-day exercises and patient education, on QoL, symptoms, discomfort, and sleep quality in people with OAB.

## 2. Materials and Methods

### 2.1. Study Design

A single-blind preliminary randomized controlled trial was conducted following the CONSORT statement. The study was conducted according to the guidelines of the Declaration of Helsinki and was approved by the University of Valencia Research Ethics Committee (2025-FIS-3870878), registered with ClinicalTrials.gob (NCT07019597). All participants gave written informed consent before being assigned to a group. In addition, written consent was obtained from the participants for the use of their images for scientific purposes. The experimental procedures were performed for 6 months.

### 2.2. Participants

Subjects with a primary diagnosis of OAB were referred by physical therapists and urologists to participate in the study. To be included, the participants were required to have shown an inadequate clinical response to prior pharmacological therapy, regardless of whether they had previously received active or alternative treatments. Participants were excluded if they had any contraindications for electrotherapy, including the presence of a pacemaker, mixed urinary incontinence, pregnancy, internal bleeding, poor skin condition, a phobia of electrical stimulation, or insufficient cognitive capacity to understand and complete the study procedures. Additional exclusion criteria included the presence of urinary fistula, urinary tract infections within the past 12 months, hematuria during the trial period, pregnancy or planning to become pregnant, central or peripheral nervous system disorders (e.g., multiple sclerosis, Parkinson’s disease), uncontrolled diabetes, recent or ongoing bladder Botox treatment, current use or implantation of an InterStim II device (Medtronic, MN, USA), bladder outlet obstruction, urinary retention, or treatment with multiple antidepressants, benzodiazepines, or antiepileptics. This study did not analyze variables related to medication use or comorbidities as there were exclusion criteria related to certain pathologies. All participants met these criteria and provided written informed consent prior to inclusion in the study. Participant screening was performed by an experienced researcher specializing in pelvic floor dysfunctions. An a priori power analysis was performed using the G*Power 3.1 software (Heinrich-Heine-Universität, Düsseldorf, Germany). An effect size of d = 0.25 was estimated with a 95% confidence interval and 80% statistical power, resulting in a required total sample of 44 participants. To account for an expected 10% dropout rate, the sample size was adjusted, giving a final target of 49 participants in total.

### 2.3. Randomization and Blinding

The participants were randomly allocated to one of two groups, either (i) the NESA Group (NNG) or (ii) the Tibial stimulation Group (TPG) using a computer-generated randomization list (SPSS statistical program, v26) by an independent researcher not involved in the study. The allocation process was carried out by an impartial person within the designated research team (allocation ratio 1:1). The assessor who performed the evaluations and the researcher who analyzed the data were blinded to the participants’ allocation.

### 2.4. Procedure and Intervention

The participants were randomly allocated to either the NNG or TNSG. All participants completed a total of 10 sessions twice weekly over a period of five weeks. In both groups, the sessions consisted of electrical modulation (either NESA Non-Invasive Neuromodulation or Posterior Tibial stimulation, depending on group allocation), combined with an abdominopelvic exercise program. Additionally, general educational guidance was delivered through standardized written instructions, which included dietary and social recommendations. The sessions were supervised by an experienced physiotherapist in pelvic health. The participants were assessed at the point of previous intervention (T0), immediately after the intervention (T1) and after a two-month follow-up (T2).

For the participants assigned to the NNG, the intervention was applied using NESA microcurrents with the participants in the supine position using gloves and anklets connected to a total of 24 electrodes, plus a directional electrode for global neuromodulation. The NESA device delivers low-frequency microcurrents (1.3–14.28 Hz, depending on the program) of a low intensity (0.1–0.9 mA), and low voltage (±3 V), organized in pulse of 130 ms, which are imperceptible to the patient. Different programs were applied daily. During the first three days, Program 1 (P1, 15 min), Program 2 (P2, 15 min), and Program 7 (P7, 30 min) were used and the directional electrode was located in the cervical (C7) area. From day four to six, P2 and P7 were applied for 30 min each (directional electrode in L3 location) and on the remaining days, P3 and P7 were applied for 30 min each (directional electrode located in S1–S2). The parameters of the NESA microcurrents depended on the program: P1 (3.85–7.69 Hz) was designed to modulate the central nervous system, P2 and P3 (1.14–1.96 Hz) targeted the ventral and dorsal areas, and P7 (1.92–14.29 Hz) aimed to achieve the focal inhibition of muscle tone and parasympathetic modulation [15].

The participants assigned to the TPG group received non-invasive posterior tibial nerve stimulation, also combined on the same day with exercises and general educational guidance. Stimulation was applied with the participants supine using the NEUROTRAC MYOPLUS PRO (Verity Medical Ltd, Sicilia, Italy) device for 30 min of TENS at 10 Hz and a pulse duration of 200 ms. Intensity was adjusted according to patient tolerance, with observable thumb flexion. Two 50 × 50 mm electrodes were placed along the posterior tibial nerve pathway: one on the plantar surface and one near the medial malleolus of the same leg.

**Figure 1:**
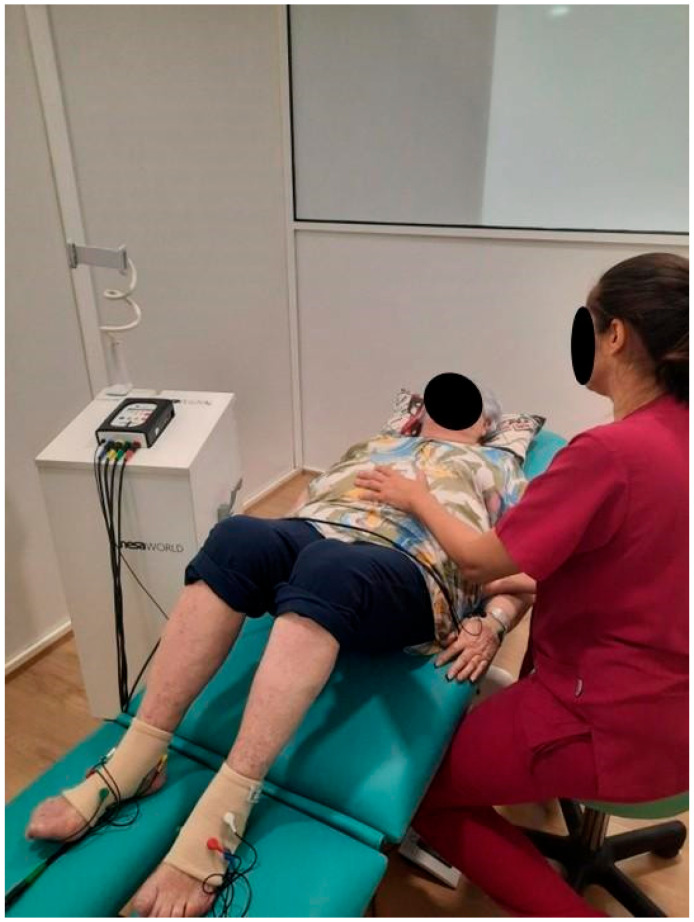
Photo of an example patient using the NESA neuromodulation during exercise (NNG). Informed consent was obtained from the patient to take the photograph.

The exercise program in both groups was developed during the electrotherapy sessions. This was explained in detail to the participants by a specialized pelvic health physiotherapist. The program consisted of abdominal-diaphragmatic breathing through tonic contractions, performing submaximal contraction (breathing in, blowing out, contracting the pelvic floor while pulling the navel in and up for approximately 7 s, which is how long the contraction lasts. Then relaxing the pelvic floor without pushing, with a rest period of approximately 4 s). This was in addition to a phasic maximal contraction (short but rapid contractions, 1 s of contraction, 5–10 repetitions, with rest periods longer than and equal to the work period, up to 10 s) over 30 min (Figure 1). A total of 3 series of tonic fibers and 2 series of phasic fibers was performed (see Supplementary Material Table S1).

### 2.5. Measure Tools

All assessments were conducted at three different times: previous intervention (T0), immediately after intervention (T1) and after a two-month follow-up (T2) by an experienced physiotherapist specializing in pelvic health. Demographic and social data was collected at baseline.

The primary outcome was urinary symptoms, focusing on urinary incontinence and its impact on QoL. Urinary symptoms were assessed using validated disease-specific questionnaires. The International Consultation on Incontinence Questionnaire-Short Form (ICIQ-SF) evaluates the frequency, severity, and overall impact of urinary incontinence through three scored items and one self-diagnostic item, with higher scores reflecting more severe symptoms and greater interference with daily activities [21]. The Spanish version of the ICIQ-SF has shown high internal consistency (α = 0.87) and excellent test–retest reliability (κ = 0.80–0.86), confirming its validity and reproducibility for assessing the severity and impact of urinary incontinence in Spanish-speaking populations [21]. Additionally, the Bladder Control Self-Assessment Questionnaire (B-SAQ), consisting of eight items divided into two subscales scored from 0 to 3, was used to assess bladder symptoms and discomfort, providing a quantitative measure of functional impairment related to pelvic floor dysfunction [21]. The Spanish adaptation of the B-SAQ has demonstrated good internal reliability (α = 0.89) and a high correlation with the King’s Health Questionnaire (r > 0.75), validating it as a useful and understandable tool for screening and monitoring urinary symptoms in adults [22].

Secondary outcomes included overall QoL, assessed using the 36-Item Short Form Health Survey (SF-36) which evaluates eight domains: physical functioning, physical role, body pain, general health, vitality, social functioning, emotional role, and mental health [23]. In addition, we evaluated sleep quality using the Pittsburgh Sleep Quality Index (PSQI), which analyses seven components: subjective sleep quality, sleep latency, sleep duration, sleep efficiency, sleep disturbances, the use of sleep medication, and daytime dysfunction. The PSQI has good applicability and the Cronbach’s alpha coefficient was 0.680 [24].

Treatment satisfaction was measured via a numeric rating scale from 0 (“not satisfied”) to 10 (“very satisfied”), capturing the participants’ subjective appraisal of the intervention. Finally, exercise adherence and adverse effects were assessed at two months through a participant self-report, providing information on the feasibility, compliance, and safety of the intervention.

### 2.6. Data Analysis

The statistical data analysis was performed using statistical SPSS software version 25.0 (SPSS Inc., Chicago, IL, USA). The normality of the variables was evaluated using the Shapiro–Wilk test. The descriptive statistics are presented as mean (standard deviation), median (25–75th percentile) or frequencies (percentage) as appropriate. For the inferential analysis to compare the two groups and across three different times (T1, T2 and T3), two-way mixed ANOVA was conducted. When the ANOVA models indicated significant differences in the main effects, Bonferroni’s correction was applied to avoid type I errors in the multiple comparisons. The α level was set at 0.05 for all tests.

Additionally, ANCOVA was performed for the variables of interest to control for potential confounders, ensuring that group differences were not influenced by these factors.

The effect sizes for the study variables were measured using Cohen’s d and r. According to Cohen’s d, the effect sizes were categorized as small (0.20 to 0.49), medium (0.50 to 0.79), or large (greater than 0.80).

## 3. Results

### 3.1. Sample

**Figure 2:**
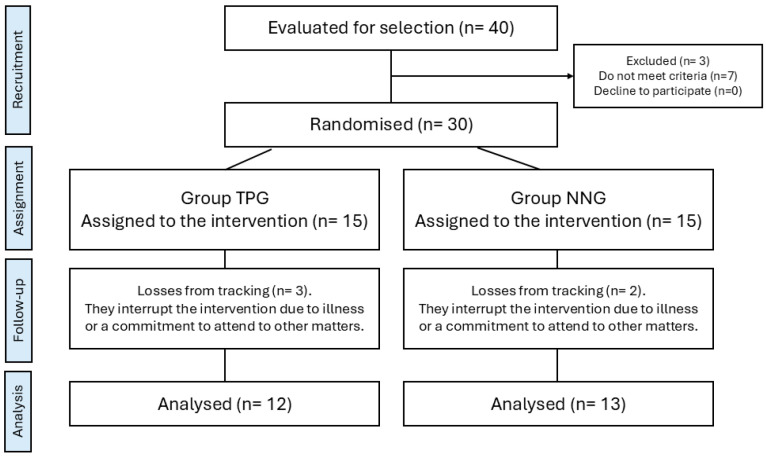
Consort flow diagram of the sample.

A total sample of 25 participants with OAB (24 women) between 33 and 85 years old (mean age 55, 84 ± 16.2) took part in this study. The participants were allocated either to the NNG (n = 13) or the TPG (n = 12). The flow diagram depicts the recruitment of subjects in this trial (Figure 2). Regarding the descriptive variables at baseline, statistically significant differences were found for age, BMI and employment status (*p* < 0.05) (Table 1). All participants completed the prescribed 10 sessions with 100% adherence and no dropouts. No adverse effects were reported.

### 3.2. Urinary Symptoms

Regarding incontinence symptoms and severity, as indicated by the ICIQ-SF scores, within-group analyses showed significant differences in both intervention groups between baseline (T1) and immediately post-intervention (T2) (NNG: 7.75 to 4.08 points; TPG: 10.08 to 6.58 points), and between baseline and the 2-month follow-up (NNG: 7.75 to 3.91 points; TPG: 10.08 to 7.41 points). However, only the NNG reported a significant reduction in urinary symptoms (*p* = 0.05) at the 2-month follow-up (T3). Across the remaining follow-up assessments, although no statistically significant differences were detected between groups, the NNG group consistently showed lower mean scores, suggesting a milder degree of urinary symptomatology (3.91 points vs. 7.41 points for NNG and TPG, respectively).

When analyzing bladder control using the B-SAQ questionnaire, the within-group analyses revealed significant improvements in both intervention groups between baseline and immediately post-intervention (T1–T2) (Table 2) and between baseline and the 2-month follow-up (T1–T3). There were no significant differences between-groups at T2 and T3 (*p* > 0.05).

ANCOVA was performed to control for the potential influence of age, BMI and employment status on both outcome measures (ICIQ-IU and B-SAQ). For the ICIQ-IU, the analysis showed that only BMI demonstrated an influence in the tibial posterior Group (TPG). The comparison between T1 and T3 was initially significant but when BMI was included as a covariate, this effect was not statistically significant (*p* = 0.76, 95% CI 0.213 to 5.49). Age did not contribute significantly to the model. For the B-SAQ, none of the covariates (age, BMI) showed significant effects

ANCOVA was performed to control for the potential influence of age, BMI, and employment status on both outcome measures (ICIQ-IU and B-SAQ). For the ICIQ-IU, the analysis showed that only BMI demonstrated an influence in the posterior tibial group (TPG). The comparison between T1 and T3 was initially significant; however, when BMI was included as a covariate, this difference was no longer statistically significant (*p* = 0.76, 95% CI 0.213 to 5.49). Age did not contribute significantly to the model. Similarly, when employment status was included as a covariate, no significant differences were observed between groups at T3 (*p* = 0.74, 95% CI −7.099 to 7.99). For the B-SAQ, none of the covariates (age, BMI, or employment status) had a significant effect. These results suggest that the initially observed differences in ICIQ-IU scores between groups at T3 may have been influenced by BMI, whereas age and employment status did not significantly affect the outcomes, and that B-SAQ outcomes appear independent of the covariates considered.

### 3.3. Quality of Life and Sleep Quality

Regarding QoL measured using the SF-36 questionnaire, within-group analyses showed significant improvements only for the NNG group between T1–T2 (*p* < 0.001) and T1–T3 (*p* < 0.001), with progressively better scores over time (Table 3). Similarly, sleep quality measured with the PSQI significantly improved in the NNG at T2 and T3 compared to T1 (*p* < 0.001 and *p* < 0.001, respectively). However, no significant between-group differences were found in either QoL or sleep quality at T2 and 3 (*p* > 0.05). Additionally, ANCOVA was performed to assess the potential influence of age, BMI, and employment status on PSQI and the SF-36 scores. The analysis did not reveal any significant effects, both within each group and regarding the between-group comparisons.

### 3.4. Satisfaction and Adherence

The average satisfaction rating for the treatment was 7.7 out of 10, with scores ranging from 5 to 10. All participants reported no side effects (100%), suggesting excellent tolerability of the treatment. During the two-month follow-up, 61% of patients continued to perform pelvic floor exercises. Of these, 26% performed the exercise 2–3 times a week, 9% performed them daily, and 26% indicated performing them intermittently.

## 4. Discussion

The present study concluded that both the 10 sessions of NESA non-invasive neuromodulation and the 10 sessions of non-invasive posterior tibial neuromodulation led to significant improvements in discomfort and symptoms in patients with OAB. However, a more notable long-term improvement was observed in the NNG. Significant improvements were also found in both groups regarding QoL and urinary incontinence symptoms, with only the NESA non-invasive neuromodulation showing a significant long-term advantage over posterior tibial neuromodulation. Additionally, sleep quality in the NNG showed a significant improvement compared to the TPG.

QoL only showed a significant improvement in the NNG, whereas the TNS did not show any improvement. To our knowledge, there is only one randomized clinical trial with characteristics like our study. The article published by Bykoviene et al. [25] compares the effects of TNS and pelvic floor exercises in women with OAB syndrome. However, they do not compare between different types of electrotherapy.

In our study, sleep quality measured with the PSQI improved significantly in the NNG both after treatment and at follow-up. Recent literature has proposed a MCID of approximately 3 points for the PSQI in the general adult population [26,27]. The mean reduction observed in our sample reached this threshold after treatment (−3.0 points) and exceeded it at follow-up (−3.92 points), suggesting that the improvement in sleep quality may be not only statistically significant but also clinically relevant. However, no significant differences were detected between groups. Given the pilot nature of the study, these findings should be interpreted with caution and be considered a subject of future research.

From a clinical perspective based on satisfaction and information from the patients of our study, the NNG reported improvements in sleep quality, urinary continence, bowel function, and overall well-being. Only 9% of participants reported no change or the relapse of symptoms after two months.

Several published studies on the innovative NESA neuromodulation highlight its modulating effect on the ANS [15,16,19,20], which supports its potential application in disorders such as OAB. Azevedo and Medina-Ramírez (2025) [28] demonstrated that non-invasive neuromodulation with NESA microcurrents can modify the autonomic response associated with pain, showing changes consistent with a sympathetic-parasympathetic rebalancing [28]. Similarly, improvements in dysautonomia symptoms have been observed in post-COVID-19 women, confirming the treatment’s ability to influence autonomic function [28,29]. Along the same lines, Contreras et al. (2023) described a reduction in pain, sleep and OAB symptoms in patients with multiple sclerosis after the application of NESA, suggesting a mechanism of action mediated by autonomic pathways [19]. Finally, highlights in European Urology focus on the relevance of the ANS as a therapeutic target for urinary incontinence, aligning the pathophysiological hypothesis with the results obtained using NESA in different clinical contexts [17]. Focusing on OAB dysfunctions with the ANS may be key to developing new treatments to improve the condition of these patients.

The results should be interpreted with caution. Some limitations exist. Firstly, the absence of a bladder diary may have reduced the availability of objective information regarding urinary parameters. Including a voiding diary in future studies would provide more detailed and comprehensive data on the patients’ urinary behavior. No objective adherence to the exercises performed was recorded, which should be considered in future studies. Secondly, the sample size was relatively small, which may have contributed to the relatively large effect sizes observed. These reflect consistent changes among the participants but should be interpreted with caution. Thirdly, multiple statistical comparisons were made, increasing the risk of type I errors. Additionally, although repeated-measures ANOVA were used for simplicity, some model assumptions may not have been fully met. Finally, baseline imbalances in age, BMI, and employment status occurred despite randomization. Although additional analyses adjusting for age and BMI were performed, residual confounding cannot be fully excluded. The treatment-effect estimates should be interpreted with caution, as the potential for bias highlights opportunities for future methodological improvements. Although this pilot study provides promising preliminary evidence, future research should incorporate a placebo group, a voiding diary, and larger sample sizes, as well as a systematic analysis of comorbidities and medication used to validate these findings and further strengthen the evidence base.

## 5. Conclusions

Non-invasive NESA neuromodulation and the Posterior Tibial neuromodulation technique showed improvements in OAB symptoms, discomfort, and sleep quality in the short term in women with OAB yet only the non-invasive NESA group had improved QoL. Further research with larger samples is needed to assess the superiority of NESA versus Posterior Tibial neuromodulation in women with OAB.

## Figures and Tables

**Table 1:** Demographic and social data of the participants.

Outcomes Mean (SD); n (%)	NNG (n = 13)	TPG (n = 12)	*p*
Age (years)	50.38 (15.63)	61.75 (15.08)	0.04 *^a^
BMI (kg/m^2^)	24.40 (4.34)	29.60 (4.04)	0.03 *^a^
Education Level			
Basic education	5 (38.30%)	2 (16.70%)	
Higher education	5 (30.80%)	3 (25.00%)	
Bachelor’s degree	3 (23.10%)	7(58.30%)	0.22 ^b^
Employment Status			
Active	8 (61.50%)	11 (63.60%)	
Non-active	4 (30.80%)	1 (36.40%)	0.003 *^b^
Marital Status			
Married	8 (61.50%)	8 (66.70%)	
Divorced	1 (7.70%)	1 (8.30%)	
Single	1 (7.70%)	3 (25.00%)	
Widow	2 (15.40%)		0.39 ^b^

NNG: Nesa Non-invasive neuromodulation group; TPG: Posterior Tibial stimulation group; BMI: body mass index; ^a^ T-Student; ^b^ Chi-Square; * *p*: statistically significant difference (<0.05).

**Table 2:** Results of the multiple comparison analysis of urinary symptoms.

Outcomes	Group	T1	T2	T3	T1–T2 *p* [95% CI]; d	T1–T3 *p* [95% CI]; d	T2–T3 *p* [95% CI]; d
Incontinence ICIQ-SF	NNG	7.75 (1.19)	4.08 (1.00)	3.91 (1.20)	0.00 * [1.08 to 6.24]; 1.39	0.00 * [−1.19 to 6.47]; 3.21	1.00 [−2.41 to 2.74]; 0.15
	TPG	10.08 (1.90)	6.58 (1.00)	7.41 (1.10)	0.00 * [6.08 to 0.92]; 2.03	0.047 * [5.03 to 0.025]; 1.72	1.00 [1.74 to −3.41]; 0.79
	NNG vs. TPG *p* [95% CI]; d	0.18 [−5.83 to 1.17]; 1.95	0.11 [−5.63 to 0.636]; 2.33	0.05 [0.018 to 7.018]; 2.92			
Bladder control B-SAQ	NNG	14.58 (1.47)	8.16 (1.27)	4.58 (1.32)	0.00 * [2.34 to 10.50]; 4.67	0.00 * [to 5.93 to 14.06]; 7.15	0.01 * [.59 to 6.57]; 2.76
	TPG	15.16 (1.46)	8.50 (1.27)	8.08 (1.32)	0.01 * [2.59 to 10.74]; 4.86	0.00 * [3.01 to 11.15]; 5.08	1.00 [−3.41 to 1.74]; 0.32
	NNG vs. TPG *p* [95% CI]; d	0.78 [−4.88 to 3.72]; 0.39	0.85 [−4.07 to 3.40]; 0.26	0.07 [−7.38 to 0.38]; 2.65			

Data shown as mean (standard deviation). ICIQ-SF: International Consultation on Incontinence Questionnaire; B-SAQ: Bladder Control Self-Assessment Questionnaire; NNG: Nesa non-invasive neuromodulation group; TPG: Posterior Tibial group; T1: baseline; T2: post-intervention; T3: 2-month follow-up; *p* *: statistically significant difference (<0.05); 95% CI: 95% confidence interval; and d: Cohen’s d.

**Table 3:** Results of the multiple comparison analysis of Quality of life and Sleep quality.

Outcomes	Group	T1	T2	T3	T1–T2 *p* [95% CI]; d	T1–T3 *p* [95% CI]; d	T2–T3 *p* [95% CI]; d
Quality of life SF-36	NNG	65.57 (5.28)	76.98 (3.28)	82.10 (3.54)	0.00 * [−21.56 to −3.72]; 2.59	0.01 * [−1.92 to −20.36]; 3.67	1.00 [−5.02 to 7.74]; 1.50
	TPG	73.41 (4.94)	77.65 (3.07)	79.60 (3.31)	0.33 [−14.62 to 3.21]; 1.03	0.14 [−16.90 to−1.0]; 1.47	1.00 [−8.22 to 4.54]; 0.61
	NNG vs. TPG *p* [95% CI]; d	0.58 [−20.93 to 12.08]; 1.53	0.71 [−11.39 to 16.41]; 0.21	0.93 [−17.50 to 16.12]; 0.73			
Sleep Quality PSQI	NNG	10.25 (0.81)	7.25 (0.96)	6.33 (1.06)	0.00 * [4.40 to 1.60]; 3.38	0.00 * [1.77 to 6.09]; 4.15	0.92 [−1.36 to 3.19]; 0.91
	TPG	9.58 (0.81)	8.50 (0.96)	7.08 (1.06)	0.17 [−0.315 to 2.48]; 1.21	0.02 * [0.35 to 4.65]; 2.65	0.36 [−0.860 to 3.69]; 1.40
	NNG vs. TPG *p* [95% CI]; d	0.57 [−1.17 to 3.04]; 0.82	0.37 [−4.06 to 1.56]; 1.30	0.62 [−3.87 to 2.37]; 0.70			

Data shown as mean (standard deviation). PSQI: Pittsburgh Sleep Quality Index; SF-36: 36-Item Short Form Health Survey; NNG: Nesa non-invasive neuromodulation group; TPG: Posterior Tibial group; T1: baseline; T2: post-intervention; T3: 2-month follow-up; *p* *: statistically significant difference (<0.05); 95% CI: 95% confidence interval; and d: Cohen’s d.

## Data Availability

The data supporting the results can be made available upon request.

## Supplementary Materials

The following supporting information can be downloaded at https://www.mdpi.com/article/10.3390/jcm14248881/s1, Table S1: Exercises performed by patients.

## Abbreviations

The following abbreviations are used in this manuscript:

OAB	Overactive Bladder
NESA	Neuroestimulacion Superficial Aplicada (Spanish)
ICIQ-SF	Internation consultation on Incontinence Questionnaire-Shor Form
B-SAQ	Bladder Self-Assessment Questionnaire
SF-36	Short Form 36 Health Survey
ANS	Autonomic Nervous System
QoL	Quality of Life
PFMT	Pelvic Floor Muscle Training
TTNS	Transcutaneous Tibialis Nerve Stimulation
TPG	Tibial Posterior group
NNG	NESA Neuromodulation Group
PF	Pelvic Floor
Hz	Hertz
ms	Milliseconds
