# Supplementary material for: Non-Invasive Autonomic Neuromodulation for Overactive Bladder: A Comparative Pilot Trial of NESA and Tibial Nerve Stimulation
Source: J Clin Med. 2025 Dec 16;14(24):8881. doi: 10.3390/jcm14248881 (PMC12733723; doi:10.3390/jcm14248881)
Supplement: Supplementary file 1 [file jcm-14-08881-s001.zip › jcm-3997754-supplementary.pdf]

Journal of
Clinical Medicine

MDPI

Table S1. Exercises performed by patients.

EXCERCISES	TECHNIQUE	PATIENT INDICATION	FIBER FOCUS	TIME	REPETITIONS
1º	Abdominal-diaphragmatic breathing Chawing-in	Take a breath, blow out and contract your pelvic floor while pulling your navel in and up. Hold the contraction for approximately 7 seconds, then relax your pelvic floor without pushing, with a rest period of 4 seconds.	Tonics	30'	3X10
2º	Kegel excercises	Short but rapid contractions, 1 second of contraction. 5-10 repetitions with a 10-second rest period.	Phasics	30'	2X10

Supplementary material (S1). *Table showing the exercise routine performed with patients during electrotherapy treatment in both groups.*
